# Household chaos, family routines, and young child movement behaviors in the U.S. during the COVID-19 outbreak: a cross-sectional study

**DOI:** 10.1186/s12889-021-10909-3

**Published:** 2021-05-04

**Authors:** Chelsea L. Kracht, Peter T. Katzmarzyk, Amanda E. Staiano

**Affiliations:** Pennington Biomedical Research Center, 6400 Perkins Road, Baton Rouge, LA 70808 USA

**Keywords:** Exercise, Television, Parent, Coronavirus, Pandemic

## Abstract

**Background:**

The home environment is an important facilitator of young child movement behaviors, including physical activity (PA), sleep, and screen-time. Household chaos, characterized by crowding, noise, and disorder in the home, may hinder efforts to obtain adequate amounts of movement behaviors. The COVID-19 outbreak impacted many families, and social distancing during this time may create conditions for more household chaos. Family routines can help establish order in the home and encourage an appropriate balance of movement behaviors, such as less screen-time and more sleep. The purpose of this study was to evaluate the association between household chaos and young child movement behaviors during the COVID-19 outbreak in the United States, and the role of family routines in this relationship.

**Methods:**

A national online survey including 1836 mothers of preschoolers (3.0–5.9 years) was conducted during May 2020. Mothers reported demographic characteristics, household chaos, family routines, and the preschooler’s movement behaviors during the outbreak. Mothers completed a household chaos questionnaire and were grouped into chaos categories (low, moderate/low, moderate/high, and high) for analysis. Linear regression was used to assess the association between chaos category, family routines, and movement behaviors with adjustment for covariates.

**Results:**

Mothers were 35.9 ± 4.1 years of age, middle income (47.8%), and preschoolers were 3.8 ± 0.8 years of age. Most mothers reported their preschooler was less physically active (38.9%), slept the same amount of time (52.1%), and increased their screen-time (74.0%) after the COVID-19 outbreak. Preschoolers in the high chaos households performed less total PA (*β* = − 0.36 days/week, 95% CI:-0.62 to − 0.09, *p* = 0.008), slept less (*β* = − 0.42 h, 95% CI:-0.59 to − 0.25, *p* = 0.001) and had more screen-time (*β* = 0.69 h, 95% CI:0.45 to 0.92, *p* = 0.001) compared to those in low chaos households. In most chaos categories, having a bed-time ritual was related to more child sleep, and mothers who viewed routines as “less/not important” reported more preschooler screen-time compared to mothers who viewed routines as “very important”.

**Conclusion:**

Promoting bed-time rituals and prioritizing routines, even somewhat, may be related to an improved balance of child movement behaviors. Innovative measures are needed to support families during periods of disruption such as that experienced in the COVID-19 pandemic.

**Supplementary Information:**

The online version contains supplementary material available at 10.1186/s12889-021-10909-3.

## Background

An appropriate balance of movement behaviors, including physical activity, screen-time, and sleep, is important for child physical and mental development [1–3]. An imbalance of movement behaviors, characterized by low levels of physical activity and sleep and high levels of screen-time, may hinder cognitive development [4, 5] and promote childhood obesity [6, 7]. The World Health Organization (WHO) recently recognized the importance of movement behaviors by creating the 24-h movement guidelines for children [8, 9]. These guidelines support original recommendations from countries across the globe, including Canada [10, 11] and Australia [12], to create multi-behavior guidelines for physical activity, sedentary behavior, and sleep during the 24-h day. Despite benefits of meeting multiple movement guidelines, cross-sectional studies indicate few (5–14.9%) preschoolers (children ages 3–5 years) meet all three guidelines, mainly driven by excessive sedentary screen-time [13, 14]. Identifying avenues to promote adequate movement behaviors can help support healthy child development.

An important component of preschooler behaviors, particularly sleep and screen-time, is the home environment [15, 16]. Accordingly, a risk factor for poor cognitive development [17] and early childhood obesity [18] is daily disruptions within the home environment or “household chaos” [19]. Household chaos encompasses high levels of household unpredictability, noise, and crowding [20]. Brief sessions of household chaos may occur in the home, but consistent exposure to household chaos may have a long-term impact on child health behaviors. Preschoolers in higher chaos households have more screen-time [21] and less sleep compared to lower chaos households [21–23]. Family routines may be a promising avenue to address household chaos as routines directly contribute to consistent amounts of behavior each day and promote biological rhythms [6]. For example, reducing screen-time before bed-time can lead to additional sleep [24, 25]. Therefore, changing family routines within a chaotic household may impact preschooler movement behaviors.

In March 2020, the WHO characterized the novel coronavirus SARS-CoV-2 (COVID-19) as a global pandemic because COVID-19 had reached many countries worldwide [26]. As COVID-19 is highly contagious, many countries encouraged their residents to practice social distancing, which included people residing within their home and limiting social gatherings [27]. Social distancing practices may result in more people residing within the home for longer periods of time and changes to work schedules, which may lead to added disarray, noise, and crowding within the home. Therefore, household changes from social distancing may contribute to more household chaos. Studies in Italian school-age children (age 6–18 years) and Canadian school-age children (ages 5–17 years) both reported children engaged in less physical activity and more sleep and screen-time during their stay at home period [28, 29]. Social distancing and potential chaos may influence preschoolers differently, as they are not subject to traditional school influences on sleep such as an early and defined school start-time [30], and require additional parental care compared to older children.

The COVID-19 outbreak provides a unique opportunity to assess household chaos, preschooler movement, and the role of family routines. The purpose of this study was to evaluate the relationship between household chaos and preschooler movement behaviors and examine the role of family routines. The primary hypothesis was that children in high chaos household will have more screen-time and less overall sleep and physical activity compared to those in lower chaos households.

## Methods

### Participants

This study was conducted using an online survey between May 1 to May 24, 2020, in the United States (US). The survey used was specifically developed for this study (Supplementary File 1). Individual state governments guided social distancing guidelines, and this period encompassed the end of most states’ stay at home orders and the beginning of “Phase 1″ or early transition periods. A convenience sample was pursued as the virus’s highly contagious properties precluded in-person recruitment. This study aimed to recruit 2500 participants, estimating that 70% would complete the entire survey (1750), and this amount (1750) would be able to detect differences in child movement behaviors individually (physical activity, sedentary screen-time, and sleep) between low and high household chaos categories based on previous literature [21]. Mothers were specifically recruited as a secondary aim of this study was to examine household chaos and maternal health behaviors, to align with previous literature of mother and child physical activity [31] and the differential impact of the COVID-19 outbreak on mother’s work-life balance [32]. Mothers of preschoolers were recruited through paid social media advertisements (i.e. Facebook), email listservs, and word of mouth. Advertisements disclosed that this study was on household dynamics and family health during the outbreak. Mothers were eligible if they had a child between the ages of 3.0–5.9 years who lived with them more than 50% of the time and lived in the US. Mothers who were younger than 18 years of age were excluded. Pennington Biomedical Research Center’s Institutional Review Board approved the study (2020–018). This study follows the STROBE guidelines for reporting of observational studies (Supplementary Table 1).

### Procedure

Potential participants accessed a secure website where they completed an online consent, screening questionnaire, demographic information, and surveys [33]. If mothers had multiple children between the ages of 3.0–5.9 years, they were asked to select one child and complete the survey once for that child. Mothers were asked to confirm their answers to four separate questions during the survey for quality assurance. Mothers who completed the entire survey were given an option to provide their email address for an opportunity to receive one of twenty US $50 checks.

### Demographic and COVID-19 factors

Mothers reported their age, ethnicity, household size, employment prior to the outbreak, and household income prior to the outbreak. In separate questions, mothers were asked to classify their current stress and stress during the last calendar year (January–December 2019) on a scale of 1–10 [34]. Mothers also reported the preschooler’s age and sex, current non-parental care status, stay at home order status, number of essential workers and health care workers in the household, teleworking status, and change in their employment and household income since the outbreak.

### Household Chaos and family routines

Household chaos was assessed using the Confusion, Hubbub, and Order Scale (CHAOS) questionnaire, to capture daily disruption or environmental confusion within the home setting. This questionnaire includes 15 questions involving disturbance, organization, and noise levels in the home, and has been previously validated in mothers of young children [20]. An example question is “No matter what our family plans, it usually doesn’t seem to work out.” Response options range from “very much like your own home” to “not at all like your own home” and are scored 1–4, respectively, with reverse coding for eight questions. Scores range from 15 to 60, with higher scores indicating a more chaotic environment. Households were grouped into chaos categories (low: < 25, moderate/low: 25–30, moderate/high: 31–35, high: > 35) similar to other research [21]. Mothers were asked how noise and the number of people within the household changed since the outbreak.

Family routine was assessed using questions related to bed-time routines and other routines within the home. Mothers were asked two questions related to having a bed-time ritual (options: yes or no) and whether the preschooler had “gone to bed at the same time each evening” (options: yes or no) [35]. These questions have been validated within a preschool-age cohort [35]. Mothers were asked how important it was to them to keep a routine prior to the outbreak (options: very, mildly, or not important) and since the outbreak (options: very, somewhat, less, or not important). Options “mildly” and “not important,” along with “less” and “not important”, were combined for analysis due to low responses to “not important.”

### Movement behaviors

The 24-h movement guidelines stipulate daily amounts of physical activity, sedentary behavior, and sleep based on child age [9, 10]. For children between ages 3–4 years, these amounts include ≥180 min/day of any intensity of physical activity (i.e. total physical activity) which includes ≥60 min of moderate-to-vigorous physical activity (MVPA), ≤1 h/day of sedentary screen-time, and 10–13 h/day of sleep [9]. For children ages 5–17 years, the guidelines include ≥60 min/day of MVPA, ≤2 h/day of sedentary screen-time, and 8–11 h/day of sleep [10]. This study assessed child total physical activity, MVPA, sedentary screen-time, and sleep. Currently, there is no universal 24-h movement questionnaire for children, thus questions from national surveys and other movement behavior investigations were used to assess movement behaviors.

Preschooler physical activity was assessed using two separate questions. To assess MVPA, mothers were asked how many days the preschooler was physically active for at least 60 min/day during the last seven days, including physical activity that “increased his/her heart rate and made him/her breathe hard some of the time”, with response options in one day increments from 0 to 7 days. This question is based on parental report of child MVPA in the National Health and Nutritional Examination Survey (NHANES) [36]. For total physical activity, mothers were asked to report how many days their preschooler engaged in any level of physical activity for at least several hours per day during the last seven days with the same response options [37]. This question was based on other parental report of total physical activity to assess 24-h movement guideline compliance [37], and was originally adapted from NHANES [36] and other validated self-report questionnaires of total physical activity [38–40]. Examples were provided of physical activity, including walking, dancing, leisurely riding a bike, and jumping jacks. Mothers compared their child’s current physical activity to the time before the outbreak.

Sleep was assessed by mother report of the preschooler’s sleep habits over the typical 24-h day since the outbreak. In separate questions, mothers reported their child’s usual amount of overnight and daytime (naps) sleep with options in 1-h increments, including a “0” option for nap, which were summed for a total sleep duration. These questions are based on components of the Child Sleep Health Questionnaire, which has been validated in school-age children [41]. Mothers compared their child’s current sleep and nap patterns to the time before the outbreak.

Mothers reported their child’s sedentary electronic screen device use over an average 24-h period since the outbreak. Mothers were asked to include smartphone, tablet, video game, and watching television or videos on internet while the child was sitting or lying down. Options ranged from none, less than one hour (treated as 0.5 h in analysis), then in one-hour increments (i.e. 1 h, 2 h, etc.) to “9 or more hours” (treated as 9 in analysis). Screen-time questions are based upon screen-time assessment recommendations from the Australian 24-h movement guidelines [12], which has the same recommendations of sedentary screen-time as the 24-h movement guidelines [9]. Mothers were asked to classify their child’s current screen-time use compared to before the outbreak.

### Data analysis

Participants with complete data for all measures (demographics, household chaos, family routines, and child movement) were included in analysis. Household chaos scores were compared within reported COVID-19 factors (current non-parental care, stay at home order, essential workers, health care workers, teleworking status, employment changes, and income changes) using one-way Analysis of Variance (ANOVA). If household chaos score differed within the COVID-19 factor, then the COVID-19 factor was included for adjustment.

Children’s movement behaviors were categorized by their age-specific recommendations, and household chaos scores were grouped into categories. Differences in demographics across household chaos categories were compared using chi-square analysis and one-way ANOVA. One-way ANOVA was also used to compare household chaos scores by reported change in movement behavior, and movement behaviors by household chaos category. Multivariable linear regression models were used to assess the association between household chaos category with preschooler movement behaviors (as dependent variables) with adjustment for preschooler age, sex, ethnicity, maternal age, prior household income, maternal employment prior to outbreak, household size, number of children in the household, mother’s current stress, US state of residence, identified COVID-19 factors, and the other movement behaviors (e.g. MVPA was adjusted for child total physical activity, sleep, and screen-time) based on prior literature [21]. Analyses were repeated with household chaos as a continuous variable. Multivariable linear regression was also used to assess the relationship between family routines (bed-time ritual, consistent bed-time, prior routines, and current routines) and movement behaviors with adjustment for the same covariates in individual models. Models were then stratified by household chaos category to examine the association between collective routines and each movement behavior found significant in individual models. Significance was set at *p* < 0.05, and all analysis were conducted with SAS 9.4 software (Cary, N.C.).

## Results

In total, 2635 participants completed the consent; 2334 were eligible; 2148 completed at least one question in the study; and 1915 completed all questionnaires (82.0% of eligible). After removing improbable answers (*n* = 2) or those who “did not know” for physical activity variables (*n* = 22) and those who missed three or more of four quality checks (*n* = 55), 1836 were included in analysis. Median time to complete the survey was 16.5 min. Compared to those not included, more of those included were employed (59.6% vs 52.2% not included) and keeping their child home (30.5% vs. 24.9% not included). There were no other differences in exposure, outcomes, or covariates between those included and not included.

Many mothers reported they were White (90.5%) and few were African American (3.2%) or Other ethnicity (6.3%), and most had an income between $70,000–$139,999 (47.8%, Table 1). All 50 states were represented, and half of mothers reported being in a stay at home order (50.2%) while the other half were transitioning out of a stay at home order (42.9%) or other phase (6.9%). Some reported there was a health care (17.2%) or essential worker (35.8%) in the household. The average household chaos score was 31.1 (SD: 7.4), indicating moderate-to-high chaos.

**Table 1:** Descriptive characteristics of sample (*n* = 1836)^

	Household Chaos Category				
Variable	Low (< 25) *n* = 381	Moderate/Low (25-30) *n* = 432	Moderate/High (31-35) *n* = 502	High (> 35) *n* = 521	*p*-value
Child Age, mean (SD), y	3.9 (0.8)	3.9 (0.8)	3.8 (0.8)	3.9 (4.0)	0.09
Child Sex, n (%), Male	173 (45.4)	206 (47.8)	247 (49.2)	269 (51.6)	0.29
Maternal Age, mean (SD), y	35.9 (4.2)	35.7 (3.9)	35.5 (4.1)	36.1 (4.0)	0.20
Maternal Ethnicity, n (%)					0.001*
White	324 (85.2)	400 (92.2)	461 (91.8)	475 (91.2)	
African American	28 (7.4)	7 (1.6)	10 (2.0)	14 (2.7)	
Other	29 (7.4)	25 (5.8)	31 (6.2)	32 (5.1)	
Maternal Hispanic Ethnicity, n (%)	26 (6.8)	24 (5.5)	30 (5.9)	30 (5.7)	0.64
Maternal Employment prior to outbreak, n (%)					0.04*
Full-time (30h hours/week)	202 (53.0)	271 (62.7)	291 (58.0)	330 (63.3)	
Part-time (< 30 h/week)	78 (20.5)	77 (17.8)	96 (19.1)	89 (17.1)	
Unemployed/Retired	101 (26.5)	84 (19.5)	115 (22.9)	102 (19.6)	
Household Income prior to Outbreak, n (%)					0.88
< $29,999	13 (3.4)	11 (2.5)	12 (2.2)	13 (2.5)	
$30,000–$69,999	66 (17.3)	67 (15.5)	94 (18.8)	82 (15.8)	
$70,000–$139,000	184 (48.3)	207 (48.0)	230 (45.9)	257 (49.3)	
$140,000 or more	104 (27.3)	130 (30.0)	147 (29.3)	145 (27.8)	
Prefer not to answer	14 (3.7)	17 (4.0)	19 (3.8)	24 (4.6)	
Number in Household Size, mean (SD)	3.9 (0.9)	4.1 (1.0)	4.2 (1.0)	4.4 (1.1)	< 0.001*
Number of Children in Household, mean (SD)	1.9 (0.9)	2.1 (1.0)	2.2 (0.9)	2.4 (1.0)	< 0.001*
Mother’s Current Stress, range 1–10, mean (SD)	5.7 (2.0)	6.5 (1.8)	7.1 (1.6)	7.9 (1.5)	< 0.001*
Region, n(%)					0.34
Midwest	89 (23.3)	92 (21.2)	122 (24.3)	126 (24.1)	
Northeast	73 (19.1)	109 (25.2)	121 (24.1)	140 (26.8)	
Southeast	124 (32.5)	128 (29.6)	142 (28.2)	141 (27.0)	
Southwest/West	95 (24.9)	103 (23.8)	117 (23.3)	114 (21.8)	
*24-h movement guidelines Met*					
Physical Activity, n (%)	124 (32.5)	111 (25.7)	111 (22.1)	104 (20.0)	0.001*
Sedentary Screen-time, n (%)	69 (18.1)	49 (11.3)	32 (6.4)	41 (7.9)	< 0.001*
Sleep, n (%)	342 (89.7)	386 (89.3)	441 (87.8)	416 (79.8)	< 0.001*
Number of Guidelines, n (%)					< 0.001*
0	20 (5.3)	31 (7.2)	45 (9.0)	87 (16.7)	
1	215 (56.4)	270 (62.5)	339 (67.5)	317 (60.8)	
2	118 (31.0)	117 (27.0)	109 (21.7)	107 (20.5)	
3	28 (7.3)	14 (3.3)	9 (1.8)	10 (2.0)	
*Family Routines*					
Bed-time Ritual, n (%)	363 (95.4)	404 (93.5)	467 (93.0)	473 (90.8)	0.06
Consistent Bedtime, n (%)	322 (84.5)	347 (80.3)	378 (75.3)	375 (72.0)	0.001*
Routine Before Outbreak, n (%)					0.001*
Very important	259 (68.0)	259 (60.0)	283 (56.5)	293 (56.3)	
Mildly/Not important	122 (32.0)	173 (40.0)	219 (44.5)	228 (43.7)	
Routines Since the Outbreak, n (%)					< 0.001*
Very Important	184 (48.4)	149 (34.4)	143 (28.9)	138 (26.5)	
Somewhat important	155 (40.8)	202 (46.8)	240 (47.9)	220 (42.3)	
Less/Not important	41 (10.8)	81 (18.8)	119 (23.2)	163 (31.2)	

^Assessed using chi-square or fisher analysis or One-way ANOVA, *p* < 0.05*

Overall, some mothers reported their preschooler performed 60 min of MVPA (30.4%) or several hours of physical activity (41.7%) every day over the last seven days, though few mothers reported their child met the sedentary screen-time guideline (children ages 3–4 years: 8.3%; children aged 5 years: 16.5%). About one quarter (23.2%) of children ages 3–4 years engaged in physical activity for at least several hours and MVPA for ≥60 min on all seven days over the last week. A similar proportion of children ages 5 years (28.1%) performed MVPA for ≥60 min on all seven days over the last week. Most children met the sleep guideline (children ages 3–4 years: 86.2; children ages 5 years: 86.4%). Based on age-appropriate guidelines, 3.3% of the sample met their respective movement behavior guidelines (children ages 3–4 years: 2.9%; children ages 5 years: 4.5%).

Most mothers reported that routines were “very important” prior to the outbreak (59.6%), but many reported routines were “somewhat important” since the outbreak (44.5%). Most mothers reported noise had increased since the outbreak (64.8%) but no change in the amount of people within the household (70.7%). Those whose child were not enrolled in non-parental care prior to the outbreak had less chaos compared to all other current non-parental care options, except for those with full-time non-parental care (Supplementary Table 2). Mothers teleworking full-time and whose household income had decreased reported more chaos compared to other respective options (Supplementary Table 2). Maternal telework status, non-parental care status, and income changes were retained as covariates in adjusted models.

### Household Chaos and preschooler movement behaviors

**Fig. 1:**
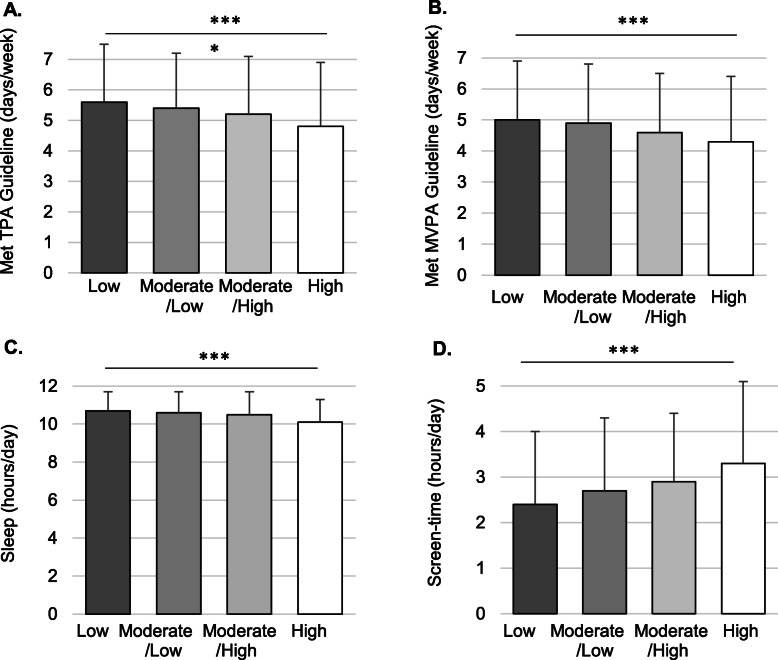
Preschooler Movement Behaviors by Household Chaos Categories^. ^Assessed using One-way analysis of variance for overall difference amongst quartiles; TPA = Total Physical Activity; MVPA = Moderate-to-vigorous physical activity; ***p < 0.001 overall difference

As shown in Fig. 1, preschooler movement behaviors significantly differed by household chaos category (*p* < 0.001 for overall difference). The proportion meeting individual movement behavior guidelines and total number of guidelines met differed by household chaos category as well (Table 1). Across household chaos categories, the guideline with the lowest adherence was the sedentary screen-time guideline. Just 7.3% of children in the low household chaos category met all three guidelines, whereas 2.0% of children in the high household chaos category met all three guidelines.

Most mothers reported their preschooler’s movement behaviors had changed in some way since the outbreak, specifically that their child was less physically active (38.9%), slept the same amount of time (52.1%), and increased their screen-time (74.0%). Those whose child had decreased physical activity, slept shorter or had poorer sleep, and had more screen-time reported more household chaos compared to most other response options in their respective category (Supplementary Table 2).

After adjustment for covariates, household chaos was still related to each movement behavior (Table 2). Compared to the low chaos category, children in the high chaos category had less total physical activity (*β* = − 0.36 days, 95% CI: − 0.62, − 0.09) and sleep (*β* = − 0.42 h, 95% CI: − 0.59, − 0.25) but more screen-time (*β* = 0.69 h, 95% CI: 0.45, 0.92). Moderate/high (*β =* 0.49 h, 95% CI: 0.27, 0.71) and moderate/low (*β =* 0.35 h, 95% CI: 0.11, 0.55) household chaos categories had higher child screen-time compared to the low chaos category. As for routines, having a bed-time ritual was positively associated with children’s MVPA and sleep and negatively associated with screen-time (Table 2). A consistent bed-time was related to more child sleep (*β =* 0.19 h, 95% CI: 0.06, 0.31) and less screen-time (*β =* − 0.28 h, 95% CI: − 0.45, − 0.10). Preschoolers whose mother reported routines were “less/not important” since the outbreak had less MVPA and sleep and more screen-time compared to those who said routines were “very important” (Table 2). Family routines were not related to total physical activity, and there was no difference in movement behaviors by prior routines.

**Table 2:** Fully adjusted associations among Household Chaos, Family Routines, and Child Movement (*n* = 1836)^

	Total Physical Activity		MVPA		Sleep		Screen-time	
Variable	β (95% CI)	*p*-value	β (95% CI)	*p*-value	β (95% CI)	*p*-value	β (95% CI)	*p*-value
*Household Chaos*								
Household Chaos score	−0.01 (− 0.03 to − 0.01)	0.01*	− 0.02 (− 0.03 to − 0.01)	0.01*	−0.02 (− 0.03 to − 0.01)	0.001**	0.04 (0.03 to 0.05)	0.001**
Household Chaos Category								
Low chaos (< 25)	Referent		Referent		Referent		Referent	
Moderate/Low chaos (25-30)	−0.08 (− 0.32 to 0.16)	0.51	0.002 (− 0.25 to 0.25)	0.99	− 0.03 (− 0.19 to 0.12)	0.65	0.35 (0.11 to 0.55)	0.001**
Moderate/High chaos (31-35)	− 0.18 (− 0.43 to 0.05)	0.13	−0.12 (− 0.37 to 0.13)	0.34	− 0.14 (− 0.30 to 0.01)	0.07	0.49 (0.27 to 0.71)	0.001**
High chaos (> 35)	−0.36 (− 0.62 to − 0.09)	0.008**	−0.19 (− 0.47 to 0.07)	0.16	− 0.42 (− 0.59 to − 0.25)	0.001**	0.69 (0.45 to 0.92)	0.001**
*Family Routines*								
Bed-time Ritual	−0.17 (− 0.49 to 0.14)	0.29	0.41 (0.07 to 0.74)	0.02*	0.58 (0.37 to 0.79)	0.001**	−0.66 (− 0.95 to − 0.38)	0.001**
Consistent Bed-time	0.08 (− 0.11 to 0.27)	0.40	0.13 (− 0.06 to 0.34)	0.17	0.19 (0.06 to 0.31)	0.003**	−0.28 (− 0.45 to − 0.10)	0.001**
Routines Before Outbreak								
Very important	Referent		Referent		Referent		Referent	
Mildly/Not important	−0.10 (−0.27 to 0.05)	0.18	−0.13 (− 0.30 to 0.03)	0.11	− 0.08 (− 0.18 to 0.02)	0.14	0.12 (− 0.02 to 0.27)	0.10
Routines Since the Outbreak								
Very important	Referent		Referent		Referent		Referent	
Somewhat important	0.04 (−0.13 to 0.23)	0.60	−0.19 (− 0.38 to − 0.01)	0.04*	−0.13 (− 0.25 to − 0.01)	0.03*	0.23 (0.16 to 0.39)	0.001**
Less/not important	0.03 (−0.19 to 0.25)	0.77	−0.28 (− 0.51 to − 0.04)	0.01*	− 0.29 (− 0.44 to − 0.14)	0.01*	0.64 (0.58 to 0.83)	0.001**

^^^Assessed using linear regression with adjustment for child age, sex, ethnicity, maternal age, household income prior to outbreak, maternal employment prior to outbreak, household size, number of children in household, maternal current stress, U.S. state of residence, current telework status of the mother, current non-parental care status, income change since outbreak, and the other three movement behaviors (total physical activity, MVPA, sleep hours, or screen-time); MVPA: Moderate-to-vigorous Physical Activity; total physical activity and MVPA represent days/week; sleep and Screen-time represent hours/day; *p < 0.05; **p < 0.01

### Household Chaos, family routines, and movement

Having a bed-time ritual was related to higher sleep in the moderate/low and moderate/high chaos categories and less screen-time in the high chaos category (Table 3). A consistent bed-time was related to less screen-time in the low chaos category (*β =* − 0.65 h, 95% CI: − 1.14, − 0.16). Mothers who viewed routines as “less/not important” since the outbreak reported higher screen-time for their child compared to those where routines were “very important” in the low, moderate/high, and high chaos categories (Table 3). Having a bedtime ritual was associated with more days of child MVPA in the moderate/low chaos category (*β =* 0.86 days, 95% CI: 0.09, 1.62, *p* = 0.02). There were no other associations between routines and children’s total physical activity or MVPA by chaos category. Overall, differing forms of routines were associated with beneficial child behavior depending on chaos level.

**Table 3:** Fully adjusted associations among Family Routines and Child Movement by Household Chaos Category (*n* = 1836)^

	Sleep		Screen-time	
*Low Chaos (n = 380)*	β (95% CI)	*p*-value	β (95% CI)	*p*-value
Bed-time Ritual	0.36 (−0.19 to 0.91)	0.20	0.15 (−0.67 to 0.99)	0.71
Consistent Bed-time	−0.22 (− 0.55 to 0.10)	0.17	− 0.65 (−1.14 to − 0.16)	0.001**
Routines Since the Outbreak				
Very important	Referent		Referent	
Somewhat important	−0.09 (− 0.32 to 0.13)	0.42	0.10 (− 0.24 to 0.44)	0.55
Less/not important	−0.15 (− 0.20 to 0.23)	0.42	069 (0.11 to 1.26)	0.01*
*Moderate/Low Chaos (n = 432)*				
Bed-time Ritual	0.58 (0.07 to 1.08)	0.02*	−0.18 (− 0.85 to 0.47)	0.57
Consistent Bed-time	0.04 (−0.26 to 0.34)	0.79	0.03 (−0.36 to 0.43)	0.86
Routines Since the Outbreak				
Very important	Referent		Referent	
Somewhat important	−0.03 (−0.29 to 0.22)	0.79	0.12 (−0.21 to 0.46)	0.48
Less/not important	−0.16 (− 0.50 to 0.17)	0.34	0.31 (− 0.14 to 0.75)	0.18
*Moderate/High Chaos (n = 502)*				
Bed-time Ritual	0.98 (0.54 to 1.42)	0.001**	−0.26 (− 0.83 to 0.32)	0.37
Consistent Bed-time	0.15 (−0.10 to 0.40)	0.24	−0.03 (− 0.35 to 0.29)	0.85
Routines Since the Outbreak				
Very important	Referent		Referent	
Somewhat important	−0.11 (−0.35 to 0.13)	0.38	0.39 (0.08 to 0.71)	0.01*
Less/not important	−0.21 (− 0.52 to 0.08)	0.16	0.61 (0.22 to 0.99)	0.002**
*High Chaos (n = 521)*				
Bed-time Ritual	0.14 (−0.26 to 0.53)	0.49	−0.91 (−1.47 to − 0.36)	0.001**
Consistent Bed-time	0.15 (−0.10 to 0.40)	0.24	0.07 (−0.27 to 0.43)	0.67
Routines Since the Outbreak				
Very important	Referent		Referent	
Somewhat important	−0.11 (−0.38 to 0.15)	0.40	0.03 (−0.34 to 0.40)	0.87
Less/not important	−0.15 (− 0.43 to 0.14)	0.33	0.47 (0.06 to 0.88)	0.02*

^^^Assessed using linear regression with adjustment for child age, sex, ethnicity, maternal age, household income prior to outbreak, maternal employment prior to outbreak, household size, number of children in household, mothers current stress, U.S. state of residence, current telework status of the mother, current non-parental care status, income change since outbreak, and the other three movement behaviors (total physical activity, moderate-to-vigorous physical activity, sleep hours, or screen-time) in the same model by chaos category; Sleep and Screen-time represent hours/day; *p < 0.05; *p* < 0.01**

## Discussion

The purpose of this study was to investigate the relationship between household chaos and preschooler movement behavior, along with the role of family routines. Preschoolers in higher chaos households had less physical activity and sleep and more screen-time compared to those in low chaos households. Bed-time rituals and current views on routines were related to preschooler sleep and screen-time across different chaos levels. This study contributes to the evidence that amidst a global pandemic and chaotic households, bed-time rituals and routines may help children achieve healthy levels of sleep and screen-time.

In this sample, many households were classified as moderate/high or high chaos. These estimates are slightly higher compared to another online survey of 385 preschool parents (24.9% moderate/high chaos and 24.4% high chaos) [21]. Slightly more mothers reported a bed-time ritual and less reported a consistent bed-time compared to a sample of 3316 young children (ages 2 years, 90.0 and 83.1% respectively) [35]. Many parents reported their child was less physically active, slept the same amount of time, and viewed more screens since the outbreak. These results are similar to findings in older children in Italian [28] and Canadian samples [29], though those children reported more sleep during the pandemic. Older children’s sleep may have increased from a lack of formal school start times, akin to delaying school start times [30], which is not as rigid in preschools. This imbalance of movement behaviors is particularly concerning given evidence that preschoolers who do not meet movement guidelines over the long-term experience negative consequences of higher adiposity [42], lower educational outcomes [5], and compromised physiological development [5], and the COVID-19 outbreak is creating a long-term challenge to household routines.

The relationship of high household chaos with less sleep and more screen-time was identified in other preschool samples [22, 23] and was found in the current study. This study adds new evidence that high household chaos is related to less physical activity in children. However, while family routines were related to sleep and screen-time, there was less support for family routines being related to physical activity. This finding may suggest that households that engage in routines may also engage in other health promoting behaviors (i.e. more physically active endeavors during the day), rather than a direct exchange of sleep and screen-time with physical activity. Other household-based options, such as family involvement in physical activity [43], should be explored to facilitate additional child physical activity in high chaos homes.

Mothers’ report of prioritizing routines in the past was not related to the child’s present movement behaviors, suggesting current actions are more important to movement behaviors. A bed-time ritual was related to additional sleep in all chaos categories, except the high chaos category. Evidence suggests implementing bed-time rituals in toddlers (8–18 months) results in additional sleep [44], even after three nights [16], which is promising for preschoolers in less chaotic households. Children within high chaos households may have more behavior problems [45], bedtime resistance [23], and face additional barriers to sleep beyond bed-time routines. High household chaos is related to delay discounting [46] and lower executive function in mothers [47], so more screen-time may be the result of changes in maternal priorities amongst a high chaos environment. Providing any added level of routine, even somewhat of a routine, within these high chaos homes may help reduce screen-time and promote sleep.

Strengths of the current study include representation across all 50 states, the use of validated questionnaires for household chaos, family routines and many of the child movement behaviors, and assessment of multiple family routines and movement behaviors during the unprecedented time of the COVID-19 stay-at-home period. The sample was predominately middle socioeconomic status and White, which may be expected from other studies using social media for recruitment [48]. There is conflicting literature on the link between household chaos and socioeconomic status [47, 49]. The current sample reported a higher household chaos score during the outbreak (Mean [SD]: 31.1[7.4]) compared to an investigation of low socioeconomic mothers of infants prior to the outbreak (Mean [SD]: 25.1[6.7]) [50], suggesting that households may have more chaos during the outbreak regardless of income status. Even without a decrease in household income, households may still face disruption in the home from teleworking and other occupational adjustments. This study had limitations, including the use of parent-report for movement behaviors necessitated by the stay at home order, which may result in social desirability bias. Along these lines, other limitations include using questions from different instruments, which are not specific to meeting the 24-h movement guidelines, and parents retrospectively reported information. Creation of a valid and reliable questionnaire to thoroughly assess 24-h movement guideline adherence in this age range is needed. Another limitations is that other health behaviors may play a role in screen-time and sleep patterns, including sleep arrangement and mealtime habits [42], and these behaviors may also differ among levels of household chaos. Examination of household chaos with device-based measures of physical activity, sedentary behavior, and sleep, such as accelerometry, may better assess the potential mechanisms.

The findings of the current study suggest three main considerations for policy makers, researchers, and physicians. First, these findings spur further investigation into family behaviors and child physical activity. Along with the results of the current study, a recent comprehensive evaluation found there is still incomplete evidence regarding the influence of family/peers and child physical activity [51]. Second, promoting bed-time rituals and prioritizing routines may enable more sleep and less screen-time across household chaos levels. Finally, the outbreak is providing an environment for moderate/high chaos and taking into consideration the additional challenges to the family (e.g. inability to access non-parental care, telework, and income changes) is important for effective behavior counseling.

## Conclusions

Overall, household chaos was related to preschooler movement behaviors, i.e. physical activity, sleep, and screen-time. Many mothers reported their household was moderately to very chaotic, and preschoolers within these households were less physically active, slept less, and engaged in more screen-time. Amid the COVID-19 outbreak and beyond, prioritizing bed-time rituals and family routines are important strategies to promote preschool children’s movement behaviors despite a chaotic household.

## Supplementary Information

**Additional file 1.** S1_Chaotic Household and Family Health Survey_4.15.21. Chaotic Households and Family Health Survey during the COVID-19 Outbreak Survey. This file contains all of the questions asked in the survey for the current study.

**Additional file 2: Table S1. **STROBE Statement—checklist of items that should be included in reports of observational studies. This table reports where components of the STROBE guidelines are presented within the document.

**Additional file 3: Table S2.** Household Chaos Score by COVID-19 Factors and Changes in Movement since the COVID-19 Outbreak (*n* = 1836). This table reports differences in household chaos score by COVID-19 factors and changes in child movement behaviors.

## Data Availability

The datasets used and/or analyzed during the current study are available from the corresponding author on reasonable request.

## Abbreviations

COVID-19: SARS-CoV-2
MVPA: Moderate-to-Vigorous Physical Activity
US: United States
WHO: World Health Organization
