# Supplementary material for: Household chaos, family routines, and young child movement behaviors in the U.S. during the COVID-19 outbreak: a cross-sectional study
Source: BMC Public Health. 2021 May 4;21:860. doi: 10.1186/s12889-021-10909-3 (PMC8094982; doi:10.1186/s12889-021-10909-3)
Supplement: Supplementary file 1 — Additional file 1. S1_Chaotic Household and Family Health Survey_4.15.21. Chaotic Households and Family Health Survey during the COVID-19 Outbreak Survey. This file contains all of the questions asked in the survey for the current study. [file 12889_2021_10909_MOESM1_ESM.docx]

**Supplementary File 3. Chaotic Households and Family Health Survey during the COVID-19 Outbreak Survey**

These questions are presented after the participant completes the consent form.

**Screening Questions (4 questions)**

1. Do you reside in the United States? *(Choose one)*
   1. Yes
   2. No 🡪 Screen out
2. Are you a mother to a child between the ages of 3.0 and 5.9 years? *(Choose one)*
   1. Yes
   2. No 🡪 Screen out
3. Does the child, ages 3.0-5.9 years, live with you more than 50% of the time? *(Choose one)*
   1. Yes
   2. No 🡪 Screen out
4. What is your age? *(Choose one, increments of 1 year from 18-56+, if 56+ then stop survey)*

**Demographic Questionnaire (18 questions)**

1. What state do you primarily live in? *(Choose one – List all U.S. states and Washington D.C.)*
2. Are you currently pregnant? *(Choose one)*
   1. Yes
   2. No
   3. Don’t Know
3. Are you of Hispanic, Latino, or Spanish Origin? *(Choose one)*
   1. Not of Hispanic, Latino, or Spanish origin
   2. Hispanic Mexican, Mexican American
   3. Chicano Hispanic Cuban
   4. Hispanic Puerto Rican
   5. Other Hispanic, Latino or Spanish origin
4. What race would you consider yourself? *(Select all that apply)*
   1. White
   2. Black of African American
   3. American Indian or Alaska Native
   4. Native Asian-Indian
   5. Asian – Chinese
   6. Asian – Filipino
   7. Asian – Japanese
   8. Asian – Korean
   9. Asian- Vietnamese
   10. Asian – Other
   11. Pacific Islander – Native Hawaiian
   12. Pacific Islander – Guamanian or Chamorro
   13. Pacific Islander – Samoan
   14. Pacific Islander – Other
   15. Other
   16. Don’t Know
5. What is your highest level of education completed? *(Choose one)*
   1. Less than high school
   2. Some high school
   3. High school diploma/GED
   4. Associate’s degree or 1-3 years of college
   5. Bachelor’s degree
   6. Graduate/professional degree
6. What is your current employment status prior to the COVID-19 outbreak? *(Choose one)*
   1. Employed full time (30=> hours)
   2. Employed part time ( < 30 hours)
   3. Unemployed/retired
7. What is your marital status? *(Choose one)*
   1. Married
   2. Divorced
   3. Never married
   4. Widowed Parent
   5. Other
8. Approximately how much do you weigh?
   1. ________ pounds *(free text, range 90-500 lbs)*
9. Approximately, how tall are you?
   1. ___________ feet _________ inches *(Choose one, range 4-6 feet, range 0-11 inches, 1 unit increments)*
10. How many people live in your household more than 50% of the time? (including yourself)?
    1. *(Choose one, 2-16+, 1 unit increments)*
11. How many children (persons 18 years or younger) live in your child’s household more than 50% of the time (including the child)?
    1. *(Choose one, 1-10, 1 unit increments)*
12. (BRANCHING) If #11 is greater than 1, How many other children live in your child’s household more than 50% of the time (not including the child)? Ages 0 to 2 years : *(Choose one, 0-10)*
    1. Ages 3-5 years: *(Choose one, 1-10, 1 unit increments)*
    2. Ages 6-10 years: *(Choose one, 1-10, 1 unit increments)*
    3. Ages 11-14 years: *(Choose one, 1-10, 1 unit increments)*
    4. Ages 15-18 years: *(Choose one, 1-10, 1 unit increments)*
13. What is the combined annual household income for your family (before taxes) prior to the outbreak?*(Choose one)*
    1. <$10,000
    2. $10,000-29,999
    3. $30,000-49,999
    4. $50,000-69,999
    5. $70,000-89,999
    6. $90,000-109,999
    7. $110,000-139,999
    8. $140,000 or greater
    9. Prefer not to answer

We are now going to ask about your child that is between 3.0-5.9 years old. If you have multiple children between the ages of 3.0 to 5.9 years, we ask that you choose one and answer the rest of the survey on that child.

1. What is the age of the child? _____ years *(Choose one, 3,4, or 5)*
2. What is the child’s sex? *(Choose one)*
   1. Male
   2. Female
3. Is the child of Hispanic, Latino, or Spanish Origin? *(Choose one)*
   1. Not of Hispanic, Latino, or Spanish origin
   2. Hispanic Mexican, Mexican American
   3. Chicano Hispanic Cuban
   4. Hispanic Puerto Rican
   5. Other Hispanic, Latino or Spanish origin
4. What race would you consider the child? *(Select all that apply)*
   1. White
   2. Black of African American
   3. American Indian or Alaska Native
   4. Native Asian-Indian
   5. Asian – Chinese
   6. Asian – Filipino
   7. Asian – Japanese
   8. Asian – Korean
   9. Asian- Vietnamese
   10. Asian – Other
   11. Pacific Islander – Native Hawaiian
   12. Pacific Islander – Guamanian or Chamorro
   13. Pacific Islander – Samoan
   14. Pacific Islander – Other
   15. Other
   16. Don’t Know
5. On average, how many hours per week did your child attend away from home care prior to the coronavirus outbreak in early 2020? This amount should include daycare, mother’s day outs, nannies, etc. *(Choose one)*
   1. 0-5 hours per week
   2. 5-10 hours per week
   3. 10-20 hours per week
   4. 21-30 hours per week
   5. 31- 40 hours per week
   6. 41+ hours per week
   7. None, my child is not enrolled in away from home care.

**Household changes since stay-at-home order and social distancing (13 questions)**

Now we are going to ask you about how the recent coronavirus (COVID-19) outbreak has influenced your family.

1. Has anyone within your household been diagnosed with the coronavirus? *(Choose one)*
   1. Yes
   2. No
   3. Unsure
2. How concerned are you about someone within your household being diagnosed with the coronavirus? *(Choose one)*
   1. Not concerned
   2. Mildly concerned
   3. Very concerned
3. What is the current social distancing guidance provided by your state? Social distancing includes residing in your home for the majority of the time, limiting outside travel, and restricting the amount of people who can gather together. *(Choose one)*
   1. Stay at home order, enforced
   2. Stay at home order, not enforced
   3. We are transitioning out of a stay at home order
   4. No state delivered order on social distancing.
   5. Other
   6. I don’t know
4. If “Other”, What "Other" social distancing guidelines have been provided by your region? *(free text)*
5. Are you currently practicing social distancing? Social distancing includes primarily residing in your home and limiting trips outside the home. *(Choose one)*
   1. Yes
   2. No
   3. I don’t know
6. (BRANCHING) If No or I don’t know to #5, Did you previously practice social distancing? *(Choose one)*
   1. Yes, we were social distancing but are no longer doing so
   2. No, we never practiced social distancing
   3. I don’t know
7. (BRANCHING) If Yes to #5, when did you stop practicing social distancing? *(Choose one)*
   1. Less than a week ago
   2. 1 week ago
   3. 2 weeks ago
   4. 3 weeks ago
   5. 4+ weeks ago
8. (BRANCHING) If Yes to #5, how long did you practice social distancing? *(Choose one)*
   1. 4 weeks or more
   2. 3 weeks
   3. 2 weeks
   4. 1 week
   5. Less than a week
9. How has your away from home care arrangement (i.e. from home care includes daycare, mother’s day outs, nannies, etc.) changed since the outbreak in early 2020? *(Choose one)*
   1. My provider is closed permanently
   2. My provider is closed temporarily
   3. My provider is open with reduced hours
   4. My provider is open as it was before the outbreak
   5. I did not use non-parental care
10. Considering this past week, how many hours is your child spending in away from home care? *(Choose one)*
    1. 0 hours, my child was not enrolled in non-parental care before the outbreak
    2. 0 hours, I do not have a non-parental care option right now
    3. 0 hours, I am keeping my child home
    4. 0-20 hours
    5. 20+ hours
11. How have you supported your child's learning at this time? *(Select all)*
    1. I am using online lessons provided by my non-parental care provider (such as daycare center)
    2. I am using online lessons I found on my own
    3. I am using existing equipment at home
    4. I am involving my child in my own work
    5. I am using his/her siblings to help with learning activities
    6. Other
12. If other to #11, what “other” ways have you supported your child's learning at this time? *(Free text)*
13. How has the outbreak impacted your household income? *(Choose one)*
    1. Total household income is reduced by more than half
    2. Total household income is reduced by 25-50%
    3. Total household income is reduced by <25%
    4. No change to household income
    5. Total household income has increased
    6. Prefer not to answer
14. Are you currently teleworking? Teleworking is primarily conducting your job within your home. *(Choose one)*
    1. Yes, full-time (more than 30 hours per week)
    2. Yes, part-time (less than 30 hours per week)
    3. No
15. How has your employment status changed since the outbreak? *(Choose one)*
    1. I am now employed and was not employed prior to the outbreak
    2. Unchanged
    3. I am working reduced hours
    4. I have lost my job
    5. N/A, unemployed prior to outbreak
16. How many people within your household work within the health care sector or are providing care to those affected by the coronavirus?*(Choose one)*
    1. 0 people
    2. 1 person
    3. 2 people
    4. 3 or more+ people
17. How many people within the household work within an essential business and are working outside the home? Examples include law enforcement, delivery services, and grocery services. This amount does not include health care workers. *(Choose one)*
    1. 0 people
    2. 1 person
    3. 2 people
    4. 3 or more people

**Household habits questionnaire (24 questions)**

On an average week since the outbreak, how often do you or another adult in the household: *(Choose one)*

|  | Never | 1-2 days | 3-4 days | 5-6 days | Everyday |
| --- | --- | --- | --- | --- | --- |
| 1. Watch your child participate in physical activity or sports |  |  |  |  |  |
| 1. Encourage your child to do sports or physical activity |  |  |  |  |  |
| 1. Provide transport to a place where your child can do physical activity or play sports |  |  |  |  |  |
| 1. Do a physical activity or play sports with your child |  |  |  |  |  |

On an average week since the outbreak, *(Choose one)*

|  | Never | 1-2 days | 3-4 days | 5-6 days | Everyday |
| --- | --- | --- | --- | --- | --- |
| 1. How often did your child eat breakfast? |  |  |  |  |  |
| 1. How often did your child eat at lunchtime? |  |  |  |  |  |
| 1. How often did your child have an evening meal? |  |  |  |  |  |
| 1. How often did you have breakfast around the table together with your children? |  |  |  |  |  |
| 1. How often did you have the evening meal around the table together with your children? |  |  |  |  |  |

| For each statement below, please assign a number between 1 and 4 to indicate how much each statement describes your current home environment. Please use the following scale: *(Choose one)*  1= Very much like your own home 2 = Somewhat like your own home 3 = A little bit like your own home 4 = Not at all like your own home   \|  \| Very much like your own home \| Somewhat like your own home \| A little bit like your own home \| Not at all like your own home \| \| --- \| --- \| --- \| --- \| --- \| \| 1. There are very few disturbances in our home \|  \|  \|  \|  \| \| 1. We can usually find things when we need them \|  \|  \|  \|  \| \| 1. We almost always seem to be rushed. \|  \|  \|  \|  \| \| 1. We are usually able to stay on top of things \|  \|  \|  \|  \| \| 1. No matter how hard we try, we always seem to be running late. \|  \|  \|  \|  \| \| 1. It’s a real zoo in our home. \|  \|  \|  \|  \| \| 1. At home we can talk to each other without being interrupted. \|  \|  \|  \|  \| \| 1. There is a lot of needless worrying going on in our home. \|  \|  \|  \|  \| \| 1. No matter what our family plans, it usually doesn’t seem to work out \|  \|  \|  \|  \| \| 1. It’s so noisy, you can’t hear yourself think in our home \|  \|  \|  \|  \| \| 1. I often get drawn into other people’s arguments at home \|  \|  \|  \|  \| \| 1. Our home is a good place to relax. \|  \|  \|  \|  \| \| 1. The telephone takes up a lot of our time at home. \|  \|  \|  \|  \| \| 1. The atmosphere in our home is calm. \|  \|  \|  \|  \| \| 1. First thing in the day, we have a regular routine at home \|  \|  \|  \|  \| |
| --- | --- | --- | --- | --- | --- | --- | --- | --- | --- | --- | --- | --- | --- | --- | --- | --- | --- | --- | --- | --- | --- | --- | --- | --- | --- | --- | --- | --- | --- | --- | --- | --- | --- | --- | --- | --- | --- | --- | --- | --- | --- | --- | --- | --- | --- | --- | --- | --- | --- | --- | --- | --- | --- | --- | --- | --- | --- | --- | --- | --- | --- | --- | --- | --- | --- | --- | --- | --- | --- | --- | --- | --- | --- | --- | --- | --- | --- | --- | --- | --- |

**Chaos Change Questions (7 questions)**

1. How has noise within the home changed since the outbreak? *(Choose one)*
   1. Our home is more noisy and loud compared to before the outbreak
   2. No Change
   3. Our home is quieter compared to before the outbreak
2. How has the number of people within the home changed since the outbreak? (*Choose one)*
   1. We have more people in our home compared to before the outbreak
   2. No change
   3. We have fewer people in our home compared to before the outbreak
3. How important was it to you to keep a routine within the home before the outbreak? (*Choose one)*
   1. Very Important to keep a routine before the outbreak
   2. Mildly important to keep a routine before the outbreak
   3. Not important to keep a routine before the outbreak
4. How important is it to you to keep a routine within the home since the outbreak? (*Choose one)*
   1. Very Important now to keep a routine
   2. Somewhat important now to keep a routine
   3. Less important now to keep a routine
   4. Not important now to keep a routine
5. What methods do you use to keep order and organization in your home? *(Select all that apply)*
   1. Morning Routine
   2. After school/child-care routine
   3. Evening Routine
   4. Bed-time Routine
   5. Weekly Schedule
   6. Dry Erase Board calendar
   7. Family Organization mobile phone apps (e.g. Cozi or a calendar app)
   8. Other:
   9. I don’t use any methods to keep our home organized
6. If “other”, what other methods do you use to keep the household organized? *(Free text)*
7. What barriers do you face to keeping a household routine and order within the home? *(select all that apply)*
   1. Changing job schedule
   2. Uncertainty in employment
   3. Lack of spouse/co-habitant support
   4. Lack of family support in area
   5. Child behavior problems
   6. Lack of child-care
   7. Unpredictable child-care
   8. Overcrowding
   9. Limited resources
   10. Uncertainty in future
   11. None of the above
   12. Other
8. If “other”, what other barriers do you face keeping a household routine and order within the home? *(Free text)*
9. What other comments do you have about keeping a household routine and order within the home at this time? ________ *(Free text)*

**Child Questionnaire (25 questions)**

Now we are going to ask about more information on the selected child that is between 3.0-5.9 years old.

1. Approximately what is the weight of your child? _____________ lbs *(free text, range 0-180)*
   1. Option for “I don’t know”
2. Approximately what is the height of the child? (One foot is equal to 12 inches)
   1. Feet *(Choose one, 1,2,3,4, or 5)*
   2. Inches (*Choose one, 0-11, in 1 inch increments)*
   3. Option for “I don’t know”

Physical Activity

Now we are going to ask you about your child’s daily physical activity patterns during the outbreak.

1. On an average week since the outbreak, on how many days did your child engage in light physical activity for at least several hours per day? Examples of light activity include walking, dancing, volleyball, hiking, playing catch, leisurely riding a bike, and jumping jacks. *(Choose one)*
   1. 0 days
   2. 1 day
   3. 2 days
   4. 3 days
   5. 4 days
   6. 5 days
   7. 6 days
   8. 7 days
   9. Prefer not to answer
   10. Don’t know
2. On an average week since the outbreak, on how many days was your child physically active for a total of at least 60 minutes per day? Add up all the time he/she spent in any kind of physical activity that increased his/her heart rate and made him/her breathe hard some of the time. *(Choose one)*
   1. 0 days
   2. 1 day
   3. 2 days
   4. 3 days
   5. 4 days
   6. 5 days
   7. 6 days
   8. 7 days
   9. Prefer not to answer
   10. Don’t know
3. How much time does your child spend playing outdoors during the week (Monday – Friday)?
   1. ________ hours __________ minutes *(Choose one, range 0-7 hours, range 0- 50 minutes in 10 minute increments)*
4. How much time does your child spend playing outdoors during the weekend (Saturday-Sunday)?
   1. ________ hours __________ minutes *(Choose one, range 0-7 hours, range 0-50 minutes in 10 minute increments)*

Now we are going to ask about how your child’s physical activity patterns have changed since the outbreak.

1. How would you classify your child’s current physical activity compared to before the outbreak? *(Choose one)*
   1. Increased compared to before the outbreak
   2. The Same
   3. Decreased compared to before the outbreak
   4. I haven’t noticed
2. How would you classify your child’s current outdoor time compared to before the outbreak? *(Choose one)*
   1. Spends more time outdoors now
   2. Spends about the same amount of time outdoors
   3. Spends less time outdoors
   4. I haven’t noticed

Sleep

Considering the time since the outbreak,

1. Do you have a set pattern or ritual with your child at bedtime? *(Choose one)*
   1. Yes
   2. No
2. Has your child usually gone to bed at the same time in the evening? *(Choose one)*
   1. Yes
   2. No
3. When has your child you usually gone to bed at night?
   1. ___________PM *(Choose one: range 1-12 hours, range 0-50 minutes in 10 minute increments)*
4. To confirm, your child usually goes to bed at *[cm_when_bed] : [cm_bed_min] PM* since the outbreak. Correct? If not, please update the previous question to reflect when your child usually goes to bed. *(Choose one)*
   1. Yes
   2. No
5. How long (in minutes) has it usually taken them to fall asleep each night?
   1. _______ minutes *(Choose one, range 0-100 min+, in 10-minute increments)*
6. When has your child usually gotten up in the morning?
   1. ___________AM *(Choose one: range 1-12 hours, range 0-50 minutes in 10 minute increments)*
7. To confirm, your child usually gets up at *[cm_up_bed] : [cm_up_min] AM* since the outbreak. Correct? If not, please update the previous question to reflect when your child usually gets up. *(Choose one)*
   1. Yes
   2. No
8. How many hours of *actual sleep* do you think your child got at night? (This may be different than the number of hours they spend in bed)
   1. __________ hours/night *(Choose one, range 1-16, 1-hour increments)*
9. How much time does your child spend napping during the day? __________ hours/day *(Choose one, range 0-8, 1-hour increments)*
10. How would you rate your child’s sleep quality overall? *(Choose one)*
    1. Very good
    2. Fairly good
    3. Fairly bad
    4. Very bad
11. Does your child use electronic screen devices (e.g. TV, video game, computer, tablet, or smartphone) in the 2 hours before bedtime on a daily basis? *(Choose one)*
    1. Yes
    2. No
    3. Don’t Know

Now we are going to ask about how your child’s sleeping patterns have changed since the outbreak

1. How have your child’s nap patterns changed compared to their nap patterns before the outbreak? *(Choose one)*
   1. Nap for a longer amount of time now
   2. More naps now
   3. Fewer naps now
   4. Naps are shorter now
   5. I have not noticed
   6. I don’t know
   7. N/A, my child does not nap
2. How have the child’s overnight sleep patterns changed compared to their overnight sleep patterns before the outbreak? *(Choose one)*
   1. Sleeps longer now
   2. Sleeps better now (fewer awakenings)
   3. The same
   4. Sleeps for a shorter amount of time now
   5. Sleep is worse now (more awakenings)
   6. I haven’t noticed

Digital Media

Now we are going to ask about how your child's digital media patterns since the outbreak.

1. On an average 24 hour period, how much time does your child spending using electronic screen devices (such as a smartphone, tablet, video game, or watch television or movies, videos on the internet) while they are sitting or lying down? *(Choose one)*
   1. Less than one hour
   2. 1 hour
   3. 2 hours
   4. 3 hours
   5. 4 hours
   6. 5 hours
   7. 6 hours
   8. 7 hours
   9. 8 hours
   10. 9 or more hours
   11. None, my child has not used an electronic screen device while they were sitting or lying down.
2. How would you classify your child’s current digital media habits compared to before the outbreak? *(Choose one)*
   1. Spends more time using digital media
   2. Uses more digital media devices
   3. No Change
   4. Spends less time using digital media
   5. Uses fewer digital media devices
   6. I haven’t noticed

**Mother Questionnaire (17 questions)**

Now we are going to ask you about your current health patterns since the outbreak and how they differ compared to before the outbreak.

Physical Activity

Considering the time since the outbreak

1. How many times on average do you do the following kinds of exercise for more than 15 minutes during your free time?
   1. Strenuous Exercise (Hear beats rapidly): _________________ times per week *(Choose one, range 0-15, 1 unit increments)*

Examples: running, jogging, hockey, football, soccer, squash, basketball, cross country skiing, judo, roller skating, vigorous swimming, vigorous long distance bicycling)

- 1. Moderate exercise (not exhausting):_____________ times per week *(Choose one, range 0-15, 1 unit increments)*

Examples: Fast walking, baseball, tennis, easy bicycling, volleyball, badminton, easy swimming, alpine skiing, popular and folk dancing

- 1. Mild Exercise (minimal effort): _______________ times per week *(Choose one, range 0-15, 1 unit increments)*

Examples: Yoga, archery, golf without using a cart, easy walking, bowling

1. During your leisure time, how often did you engage in any activity long enough to work up a sweat (heart beats rapidly)?
   1. Often
   2. Sometimes
   3. Never/Rarely

Now we are going to ask about how your physical activity patterns have changed since the outbreak

1. How would you classify your current physical activity compared to your physical activity before the outbreak?
   1. More active now
   2. The same
   3. Less active now
   4. I haven’t noticed

Sleep

The following questions relate to your usual sleep habits during the since the outbreak in early 2020. Your answers should indicate the most accurate reply for the majority of days and nights since that time**.**

Considering the time since the outbreak,

1. When have you usually gone to bed at night?
   1. ___________*(Choose one: AM or PM, range 1-12 hours, range 0-50 minutes in 10 minute increments)*
2. To confirm, you usually go to bed at *[mother_when_bed:value] : [mother_bed_min] [mother_ampm]* since the outbreak. Correct? If not, please update the previous question to reflect when you usually go to bed. *(Choose one)*
   1. Yes
   2. No
3. How long (in minutes) has it usually taken you to fall asleep each night?
   1. _______ minutes *(Choose one, 0-100+, in 10 minute increments)*
4. When have you usually gotten up in the morning?

a. ___________*(Choose one: AM or PM, range 1-12 hours, range 0-50 minutes in 10 minute increments)*

1. To confirm, you usually get up at *[mother_up_bed:value] : [mother_up_min] [mother_ampm_2]* since the outbreak. Correct?If not, please update the previous question to reflect when you usually go to bed. *(Choose one)*
   1. Yes
   2. No
2. How many hours of *actual sleep* did you get at night? (This may be different than the number of hours you spend in bed)
   1. __________ hours/night *(Choose one, range 0-14, 1-hour increments)*
3. How would you rate your sleep quality overall? *(Choose one)*
   1. Very good
   2. Fairly good
   3. Fairly bad
   4. Very bad
4. How often have you had trouble staying awake while driving, eating meals, or engaging in social activity? *(Choose one)*
   1. Not during the past month
   2. Less than once a week
   3. Once or twice a week
   4. Three or more times a week
5. How much of a problem has it been for you to keep up enough enthusiasm to get things done? *(Choose one)*
   1. No problem at all
   2. Only a very slight problem
   3. Somewhat of a problem
   4. A very big problem

Now we are going to ask about how your sleep patterns have changed since the outbreak

1. How have your overnight sleep patterns changed compared to your sleep patterns before the outbreak? *(Choose one)*
   1. I sleep longer now
   2. I sleep better now (better quality)
   3. No change
   4. I sleep for a shorter amount of time
   5. I sleep worse (worse quality)
   6. I haven’t noticed

Digital Media

Considering the time since the outbreak,

1. On an average 24 hour period, how much time does your child spending using electronic screen devices (such as a smartphone, tablet, video game, or watch television or movies, videos on the internet) while they are sitting or lying down? *(Choose one)*
   1. Less than one hour
   2. 1 hour
   3. 2 hours
   4. 3 hours
   5. 4 hours
   6. 5 hours
   7. 6 hours
   8. 7 hours
   9. 8 hours
   10. 9 or more hours
   11. None, I have not used an electronic screen device while I was sitting or lying down.
2. How have your digital media patterns changed compared to your digital media patterns before the outbreak? *(Choose one)*
   1. Spend more time using digital media
   2. Uses more digital media devices
   3. No change
   4. Spend less time using digital media
   5. Uses fewer digital media devices
   6. I haven’t noticed

Now we are going to ask about your health state.

On a scale from 1 to 10, where 1 means “no stress” and 10 means “an extreme amount of stress,”

1. . How much stress would you say you have experienced in the since the outbreak? *(Choose one, range 1-10, 1 unit increments)*
2. How would much stress would you say you have experienced in the year 2019 (January 2019- December 2019)? *(Choose one, range 1-10, 1 unit increments)*

**End Message**

Thank you for participating in this study. Below are a few resources for families during the COVID-19 Outbreak.

Partners for Family Health COVID-19 Resources

[Mental Health Resources for Families during the Coronavirus Outbreak | Partners for Family Health](https://partnersforfamilyhealth.org/mental-health-resources-families-coronavirus-outbreak/)

At Home Resources from Family Education

[At-Home Learning Resources for the COVID-19 Outbreak - FamilyEducation](https://www.familyeducation.com/at-home-learning-resources-for-the-covid-19-outbreak)

Pennington Biomedical Research Center Coronavirus Resource Center

[Pennington Biomedical Research Center (pbrc.edu)](https://www.pbrc.edu/coronavirus/)

If you have any questions about the study, please contact PLAYStudy@pbrc.edu. If you are interested in entering a drawing for $50.00, please enter your email address below. Selected recipients will be contacted at the end of the study. Click "Submit" to end the survey.
