# Supplementary material for: Household chaos, family routines, and young child movement behaviors in the U.S. during the COVID-19 outbreak: a cross-sectional study
Source: BMC Public Health. 2021 May 4;21:860. doi: 10.1186/s12889-021-10909-3 (PMC8094982; doi:10.1186/s12889-021-10909-3)
Supplement: Supplementary file 3 — Additional file 3: Table S2. Household Chaos Score by COVID-19 Factors and Changes in Movement since the COVID-19 Outbreak (n = 1836). This table reports differences in household chaos score by COVID-19 factors and changes in child movement behaviors. [file 12889_2021_10909_MOESM3_ESM.docx]

| Supplementary Table 2. Household Chaos Score by COVID-19 Factors and Changes in Movement since the COVID-19 Outbreak (*n*=1,836)^ | | | |
| --- | --- | --- | --- |
| **COVID-19 Factors** |  | Household Chaos Score | *p*-value |
| *Non-parental Care Status, n, mean (SD)* |  |  | <0.001*** |
| Full-time (21+ hours/week) | 119 | 30.4 (7.6) |  |
| Part-time (1-20 hours/week) | 146 | 31.3 (6.6) |  |
| Not enrolled prior to outbreak (0 hours/week) | 213 | 28.9 (7.2) |  |
| No non-parental care option (0 hours/week) | 799 | 32.0 (7.6) |  |
| Keeping child home (0 hours/week) | 536 | 30.6 (7.3) |  |
|  |  |  |  |
| *Maternal Teleworking Status, n, mean (SD)* |  |  | <0.001*** |
| Full-time | 723 | 31.7 (7.5) |  |
| Part-time | 336 | 31.1 (7.1) |  |
| No telework | 777 | 30.5 (7.5) |  |
|  |  |  |  |
| *Change in Household Income, n, mean (SD)* |  |  | <0.001*** |
| Increased | 117 | 29.9 (7.7) |  |
| No change | 1108 | 30.4 (7.3) |  |
| Decreased | 611 | 32.5 (7.6) |  |
|  |  |  |  |
| **Change in Child Movement** |  |  |  |
| *Child Physical Activity, n, mean (SD)* |  |  | <0.001*** |
| Increased compared to before the outbreak | 465 | 30.4 (7.0) |  |
| The same | 639 | 29.7 (7.1) |  |
| Decreased compared to before the outbreak | 716 | 32.6 (7.7) |  |
| I haven’t noticed | 16 | 35.7 (10.2) |  |
|  |  |  |  |
| *Child Sleep - Overnight, n, mean (SD)* |  |  | <0.001*** |
| Sleeps longer now | 253 | 30.4 (6.7) |  |
| Sleeps better now (less awakenings) | 97 | 31.9 (7.8) |  |
| The same | 957 | 29.8 (7.3) |  |
| Sleeps for a shorter amount of time now | 148 | 33.5 (7.8) |  |
| Sleep is worse now (more awakenings) | 326 | 33.6 (7.5) |  |
| I haven’t noticed | 55 | 33.6 (6.7) |  |
|  |  |  |  |
| *Child Sleep – Nap, n, mean (SD)* |  |  | 0.07 |
| Nap for a longer amount of time now | 102 | 31.8 (6.7) |  |
| More naps now | 72 | 31.3 (8.3) |  |
| Fewer naps now | 488 | 31.6 (7.3) |  |
| Naps are shorter now | 135 | 31.5 (7.6) |  |
| I haven’t noticed | 142 | 29.4 (7.2) |  |
| I don’t know | 26 | 31.5 (8.2) |  |
| My child doesn’t nap | 871 | 30.8 (7.5) |  |
|  |  |  |  |
| *Child-Device Use, n, mean (SD)* |  |  | <0.001*** |
| Spends more time using digital media | 1360 | 31.8 (7.4) |  |
| Uses more digital device devices | 151 | 31.2 (6.9) |  |
| The same | 278 | 28.2 (7.4) |  |
| Spends less time using digital media or uses fewer devices | 47 | 31.1 (7.7) |  |
|  |  |  |  |
| *^^^Assessed using One-Way Analysis of Variance, p<0.05*; **p<0.01; ***p<0.001* | | |  |
